# Gamma Knife^®^ radiosurgery for recurrent intracranial olfactory neuroblastoma (esthesioneuroblastoma): a case report

**DOI:** 10.1186/1752-1947-6-240

**Published:** 2012-08-13

**Authors:** Eduard B Dinca, Matthias W Radatz, Jeremy Rowe, Andras A Kemeny

**Affiliations:** 1 The National Centre for Stereotactic Radiosurgery, Royal Hallamshire Hospital, Glossop Road, Sheffield, S10 2JF, UK

## Abstract

**Background:**

We report the use of salvage radiosurgery to manage an aggressive olfactory neuroblastoma (esthesioneuroblastoma) with multiple recurrences and intracranial extension.

**Case presentation:**

A 43-year-old Caucasian woman presented 11 years ago with progressive nasal blockage and headaches. A necrotic polyp originating in her left middle meatus and extending to the ethmoid air cells and cribriform plate (Kadish stage C) was radically resected via a craniofacial approach. Four years later, a local recurrence extending into her left cavernous sinus was identified and deemed inoperable. She received vincristine, ifosfamide, doxorubicin and etoposide chemotherapy (with minimal benefit) and external beam radiotherapy (60Gy in 30 fractions) to her skull base. Two years later, tumour extension in her left neck was treated with radical radiotherapy. She developed visual disturbances in her left eye, which progressed to blindness in the next two years. Having exhausted chemoradiotherapy, the left cavernous sinus esthesioneuroblastoma was treated with Gamma Knife® radiosurgery 2 years ago (20Gy at 50% isodose, tumour volume 7.5cm^3^). At one year, there was dramatic reduction in the tumour and no new symptoms; however, there were new tumour foci (in her left frontal lobe and above her right orbital apex). These were again treated with radiosurgery (20Gy at 50% isodose, total tumour volume 0.67cm^3^). Repeat imaging at six months showed no further disease progression.

**Conclusion:**

Whilst rare, olfactory neuroblastoma (esthesioneuroblastoma) can present management challenges and Gamma Knife® radiosurgery may prove a useful strategy in controlling intracranial spread.

## Background

We report radiosurgical management for the intracranial component of an aggressive olfactory neuroblastoma (esthesioneuroblastoma) with multiple recurrences. This minimally invasive technique was used as a ‘last resort’ for a patient who had already exhausted the available surgical and chemoradiotherapy options.

## Case presentation

Our patient, a 43-year-old Caucasian woman, first presented 11 years ago with nasal blockage and headaches, originally thought to be due to her ongoing *in vitro* fertilization treatment. A necrotic polyp originating in her left middle meatus and extending superiorly to the ethmoid air cells and the cribiform plate (Kadish stage C) was resected through a craniofacial approach. Four years later, a local recurrence extending into her left cavernous sinus was identified. As it was deemed inoperable, chemotherapy was tried, and she received three rounds of the vincristine, ifosfamide, doxorubicin, etoposide (VIDE) regimen. This proved of minimal benefit, and external beam radiotherapy was administered to her skull base (60Gy over 30 fractions). Two years after this, tumour extension in her left neck was treated with radical radiotherapy. In the next couple of years, she developed visual disturbances in her left eye, initially as ‘zig-zag’ lights and impairment of central vision, which rapidly progressed to blindness, probably as a combined consequence of skull base disease progression and delayed side effects from multiple treatments. At this time, the progression of her intracranial disease left management teams at a loss regarding further active management.

**Figure 1:**
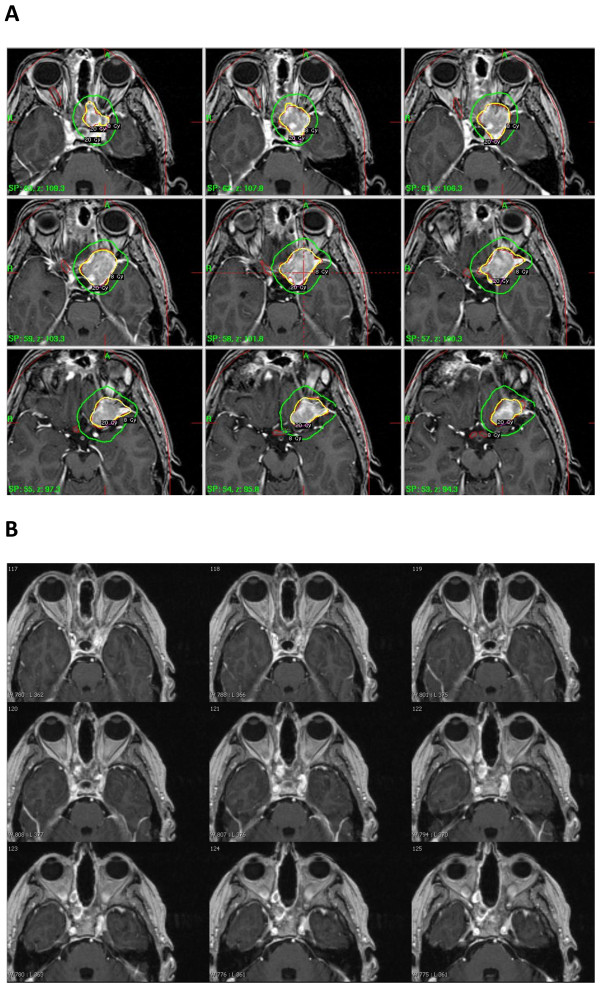
**Magnetic resonance images.** (**A)** Axial T1-weighted, contrast-enhanced magnetic resonance images at time of first treatment, showing the left cavernous sinus tumour and treatment isodoses as outlined in the Gamma Knife® planning software. **(B)** Axial T1-weighted, contrast-enhanced magnetic resonance images after 18 months.

**Figure 2:**
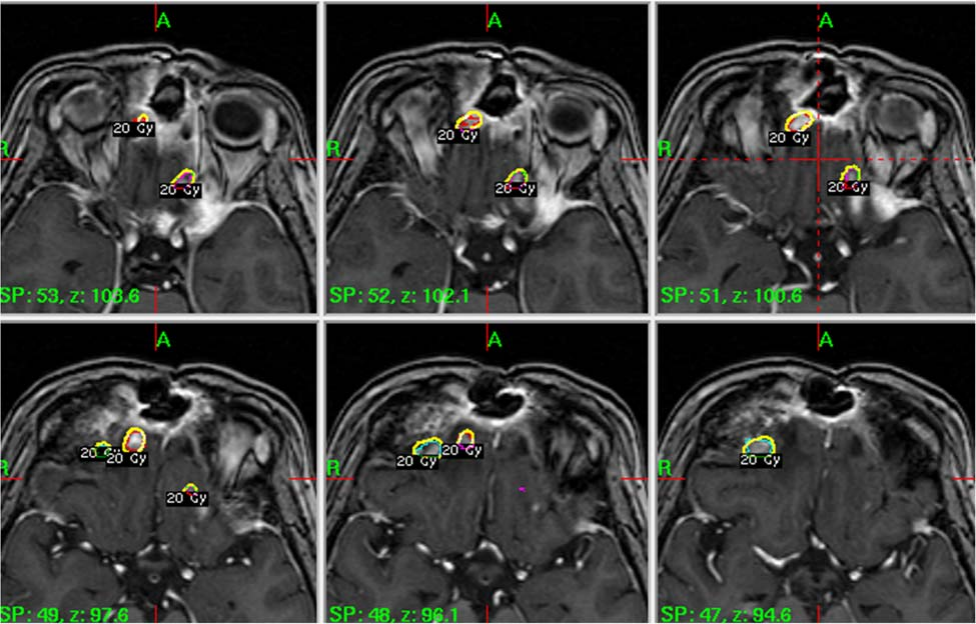
**Axial T1-weighted, contrast-enhanced magnetic resonance images one year after the first treatment, showing new, small tumour foci in the left frontal lobe and above the right orbital apex.** These were treated with a new session of radiosurgery. Treatment isodoses are shown as outlined in the Gamma Knife® planning software.

The left cavernous sinus esthesioneuroblastoma (tumour volume 7.5cm^3^) was treated with Gamma Knife® radiosurgery (GKRS) two years ago (20Gy at 50% isodose), and our patient showed excellent recovery (Figures 1A). Follow-up one year later revealed no new side effects and a dramatic reduction in the size of the treated tumour, further documented by magnetic resonance imaging at 18 months (Figures 1B). However, this one-year follow-up also showed tumour foci in the adjacent left frontal lobe and above her right orbital apex (Figures 2), which were treated with a new session of GKRS (20Gy at 50% isodose, total tumour volume 0.67cm^3^).

## Discussion

Esthesioneuroblastoma is a very rare neuroectodermal malignancy (approximate incidence one in 2.5 million), arising from specialized olfactory cells [1]. It involves the junction of the upper nasal cavity and the anterior intracranial fossa, typically extending upwards through the cribiform plate of the ethmoid to various degrees of extra-axial and brain involvement. In spite of its cell of origin, anosmia is a rather uncommon presenting symptom (5%). The most common complaints are nares blockage (70%) and epistaxis (50%), followed in no particular order by persistent rhinorrhea, watery eye through blockage of the lacrimal duct, and intracranial signs like headache and visual disturbances [1,2]. Brain imaging studies (computed tomography and magnetic resonance imaging [MRI]) typically show a dumbbell-shaped mass eroding across the cribiform plate, with homogenous enhancement (cold spots signify intratumoural necrosis). Pathology shows neuroblastoma cells arranged in a lobular architecture, within a continuum of aggressiveness from Hyams grade I to grade IV (as differentiation decreases whilst pleomorphism, mitoses and areas of necrosis progress from absent to marked) [3].

Several staging systems have been proposed. At the time of stereotactic radiosurgery, our patient’s tumour was Kadish stage C (‘spread beyond nasal and paranasal cavities’) and grade IV according to other classifications (Biller ‘unresectable tumour’; Dulguerov-Calcaterra, ‘tumour involving the brain’) [4-6].

There have been over 1000 reports of olfactory neuroblastoma since its description in 1924 [2,7]. Nevertheless, it remains exceedingly rare, and this may be the main reason why there is still no therapeutic consensus regarding the use and timing of different management options including surgery, external beam radiotherapy and chemotherapy. A report cited surgery as the only treatment, restricting radiation’s role to palliation [4], perhaps in an attempt to obviate the serious side effects incurred by radical conventional radiotherapy of the craniofacial junction, including blindness (which our patient has also experienced) [8]. Others [5,9] emphasized the complementary roles of both therapies, as the upper limit of local recurrence ranges from 33% with multi-modal management to 50% with surgery alone [2]. A series by the Mayo Clinic [10] evaluating cisplatin-based chemotherapy yielded a control rate of 30%; however, the small sample size (10 patients) did not allow any guidelines to be drawn. Currently, the five-year survival is estimated at 40% for high-grade tumours and 80% for low-grade tumours [1].

We are aware of only one other group in the world to have reported the use of stereotactic radiosurgery for olfactory neuroblastoma [2,11,12]. The Graz (Austria) group reported a series of 14 patients having received upfront bimodal treatment: endoscopic surgery for the nasal and paranasal component and GKRS for the intracranial component. Margin doses between 15 and 34Gy were used to treat tumour volumes up to 22cm^3^, achieving good long-term control with virtually no radiosurgery-related side effects. Local tumour control was 100%. While tumour recurrence at distant sites was noted in five of their patients, only one needed a craniotomy and the other four were successfully managed by a new session of GKRS.

We here report the GKRS management of recurrent intracranial disease in a patient for whom all other options - surgery, radiotherapy and chemotherapy - were deemed exhausted. The sizeable tumour (7.5cm^3^) in her left cavernous sinus showed an excellent response. The new tumour foci identified on the one-year follow-up MRI were managed by another session of GKRS, and they also appeared to be stable six months after treatment. We used a 20Gy dose as a previous report [2] noted no significant differences in outcome for marginal doses between 15 and 34Gy. Plan conformality did benefit from the fact that our patient was already blind in her left eye; that being said, we note same-dose GKRS achieves excellent results with other cavernous sinus tumours of even greater size (for example, meningiomas), for which careful planning to avoid afflicting the visual pathways has been proven a definite possibility. The very principle of GKRS, allowing delivery of a high radiation dose in a single fraction, maximizing it to the target while minimizing the radiation to surrounding tissues, ensures good local control while maintaining a benign side effect profile, preserving the best quality of life possible for an oncology patient. Moreover, and perhaps most importantly, it allows for procedure repeatability, so that regular imaging and re-treatment of any new intracranial lesions becomes an attractive option.

## Conclusions

The current standard of care - surgery and radiation - carries a high risk of morbidity and serious side effects (visual and/or cognitive), while still leaving the patient at risk for recurrence. Due to its low morbidity, non-existent mortality and reasonable side effect profile, GKRS is certainly an option to consider when dealing with intracranial esthesioneuroblastoma. While we report its value as a last resort for a patient who has already exhausted surgery and chemoradiotherapy, further experience is necessary to document its role as a valid primary option for intracranial olfactory neuroblastoma.

## Consent

Written informed consent was obtained from the patient for publication of this case report and accompanying images. A copy of the written consent is available for review by the Editor-in-Chief of this journal.

## Competing interests

The authors declare that they have no competing interests.

## Authors’ contributions

EBD was responsible for the conception and design, acquisition of data, analysis and interpretation of data, and writing the manuscript; MWR, JR and AAK have been involved in direct patient care at different time points, as well as in revising the manuscript critically for important intellectual content. AAK gave final approval of the version to be published. All authors read and approved the final manuscript.
